# Crude Petroleum in Asthma

**Published:** 1880-09

**Authors:** 


### CRUDE PETROLEUM IN ASTHMA.

M. M. Griffith, M. D., in the *Medical Record:*

It is a well-known fact that many of our most valuable medicines have been borrowed or developed from general impressions or the prevailing prejudice of the common people in some district or country. Jenner deduced an important scientific truth from the vague notions and common prejudice of the dairymen of Gloucestershire. In like manner has it been with many of the important remedies of the now extensive materia medica, which have often been in use by the common people before being investigated by the profession.

Pursuing this line of observation, we find the veterinary surgeons, farmers, and horse-jockeys now prescribing the ordinary crude petroleum as a remedy for broken wind and heaves in horses, and with astonishing success, improving the general condition of the animal, giving him a fine appearance, and removing the difficulty of breathing as if by magic; a cure which they are willing to swear is permanent, which assertion I accept with several grains of allowance. Heaves and broken wind I have always looked upon as due to emphysema, and consequently treatment must necessarily be only palliative. Crude petroleum is a stimulating antispasmodic expectorant and diaphoretic of no mean power. It seems to act by stimulating the secretions generally, especially those of the skin, and improving the digestive functions. The dose for the horse is one teaspoonful, in meal, placed well back upon the tongue two or three times a day, continued until relief is afforded.

Having seen the beneficial effects of this remedy frequently applied to the horse, I was led to experiment upon that difficult disease to cure, asthma. I used the ordinary oil in various combinations, as in syrups, emulsions, etc.; but however it might be combined, I found that it always produced a disagreeable eructation, and it was hard to induce patients to persevere in its continuance. But the semi-solid oil that accumulates on the tubing and casings of the wells, and hardens to the consistency of putty, made into pills of five grains by incorporating with some inert vegetable powder, taken every three or four hours, has afforded almost instant relief. The paroxysms will not return under its usage. It is not curative, but the patient does not suffer while taking the pills, and after a few days the spasmodic symptoms seem to pass off. Many asthmatics are affected only in the spring or fall, and after these attacks pass off they are comparatively comfortable. Nothing has afforded me as much relief in the treatment of hay fever, autumnal catarrh, or asthmatic bronchitis as these pills. The cough and dyspnœa are promptly alleviated.

I have already called the attention of the profession to the value of this remedy in pulmonary tuberculosis.—*Louisville Medical News.*

